# Current Advancement and Future Prospects in Simplified Transformation-Based Plant Genome Editing

**DOI:** 10.3390/plants14060889

**Published:** 2025-03-12

**Authors:** Xueying Han, Zhaolong Deng, Huiyun Liu, Xiang Ji

**Affiliations:** State Key Laboratory of High-Efficiency Production of Wheat-Maize Double Cropping, and Center for Crop Genome Engineering, College of Agronomy, Henan Agricultural University, Zhengzhou 450046, China; 18838917027@163.com (X.H.); dzl16536@163.com (Z.D.)

**Keywords:** plant genome editing, genetic transformation, developmental regulators, *in planta* transformation, nanomaterials, viral vector

## Abstract

Recent years have witnessed remarkable progress in plant biology, driven largely by the rapid evolution of CRISPR/Cas-based genome editing (GE) technologies. These tools, including versatile CRISPR/Cas systems and their derivatives, such as base editors and prime editors, have significantly enhanced the universality, efficiency, and convenience of plant functional genomics, genetics, and molecular breeding. However, traditional genetic transformation methods are essential for obtaining GE plants. These methods depend on tissue culture procedures, which are time-consuming, labor-intensive, genotype-dependent, and challenging to regenerate. Here, we systematically outline current advancements in simplifying plant GE, focusing on the optimization of tissue culture process through developmental regulators, the development of *in planta* transformation methods, and the establishment of nanomaterial- and viral vector-based delivery platforms. We also discuss critical challenges and future directions for achieving genotype-independent, tissue culture-free plant GE.

## 1. Introduction

In recent years, the rapid development of genetic engineering technologies has driven transformative progress in plant research. Among these, CRISPR/Cas-based genome editing (GE) technologies have significantly accelerated the dissection of gene functions and precision molecular breeding in plants due to their simplicity, efficiency, and precision [1,2]. By programming natural CRISPR/Cas systems and their derivatives, including base editors and prime editors, researchers have achieved efficient gene knockout and deletions, transcriptional regulation, base editing, and small fragment insertions or replacements in plants [3,4,5,6,7]. Despite their versatility, the reliance on a tissue culture-based transformation process, which is lengthy, labor intensive, and often genotype-dependent, has imposed a serious bottleneck for fully materializing the potential of plant GE [8,9].

**Figure 1:**
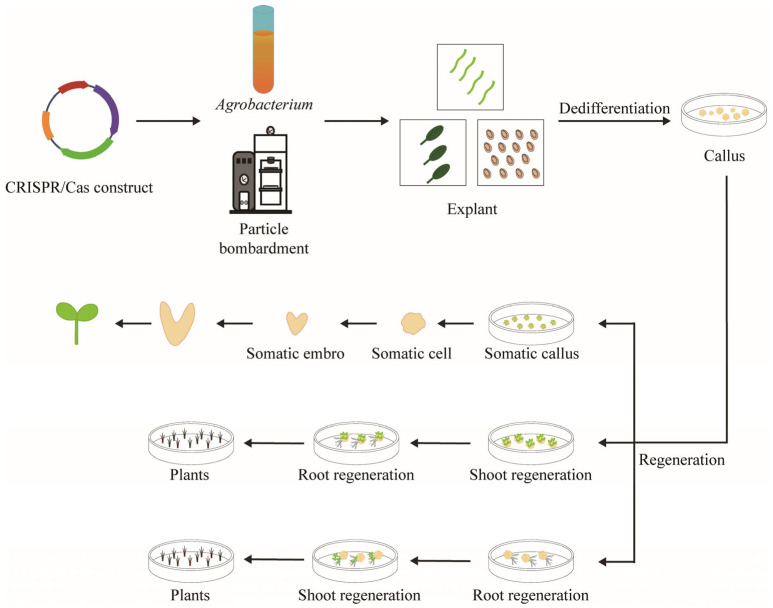
Schematic illustration of the transformation-based plant GE process. This process is fundamentally reliant on conventional genetic transformation techniques, which primarily involve the introduction of CRISPR/Cas-based GE constructs into recipient cells through either *Agrobacterium*-mediated transformation or particle bombardment. The tissue culture process consists of several critical stages, extending from the acquisition of explants to the regeneration of whole plants. Explants can be sourced from various plant tissues, including roots, stems, leaves, shoot tips, embryos, and endosperm (depicted in the upper half of the figure). During tissue culture and regeneration, the manifestation of plant cell totipotency is exemplified through organ regeneration, including *de novo* root and shoot formation, as well as somatic embryogenesis (depicted in the lower half of the figure).

The generation of edited plant mutants largely depends on traditional genetic transformation processes, primarily through *Agrobacterium*-mediated or particle bombardment-aided DNA transfer to deliver the CRISPR/Cas-based GE constructs into recipient cells [2,10], followed by obtaining complete regenerated plants through tissue culture (Figure 1) [11]. *Agrobacterium*-mediated DNA delivery is the preferred method for most plant species. This method utilizes *Agrobacterium*, a Gram-negative soil pathogen that naturally carries transgenic Ti or Ri plasmids. These plasmids can be engineered to transfer foreign DNA into plant chromosomes for stable inheritance and expression [12,13]. This method is widely favored due to its simplicity, low transgene copy number, reliable expression, and relatively low cost. In contrast, particle bombardment, also known as the gene gun method, is a physical technique that uses the force of an explosion or high-pressure gas to propel particles carrying a gene of interest into plant cells [14,15,16]. This method allows for the non-specific introduction of foreign genes into plant cells, tissues, or organs, making it easy to operate, fast in terms of delivery, and not limited by the genotype of the recipient. However, it also presents drawbacks, such as the potential to damage cells or tissues, difficulty in producing heritable mutations, and high costs [17].

To regenerate GE plants after DNA delivery, most plant species must undergo tissue culture, which is a time-consuming process that often takes months to accomplish. This process typically involves the use of expensive and environmentally harmful reagents (e.g., antibiotics and herbicides) in the growth medium to select transgenic plants and regenerate plantlets from recipient tissues (Figure 1) [18]. Moreover, low genetic transformation efficiency, along with genotype-specific limitations, remains a key bottleneck hindering the broader application of GE technologies in plants. In this review, we highlight recent progress in simplified plant GE strategies and provide future perspectives.

## 2. Strategies for Simplified Plant GE

### 2.1. Enhancing Tissue Culture Efficiency with Developmental Regulators

**Figure 2:**
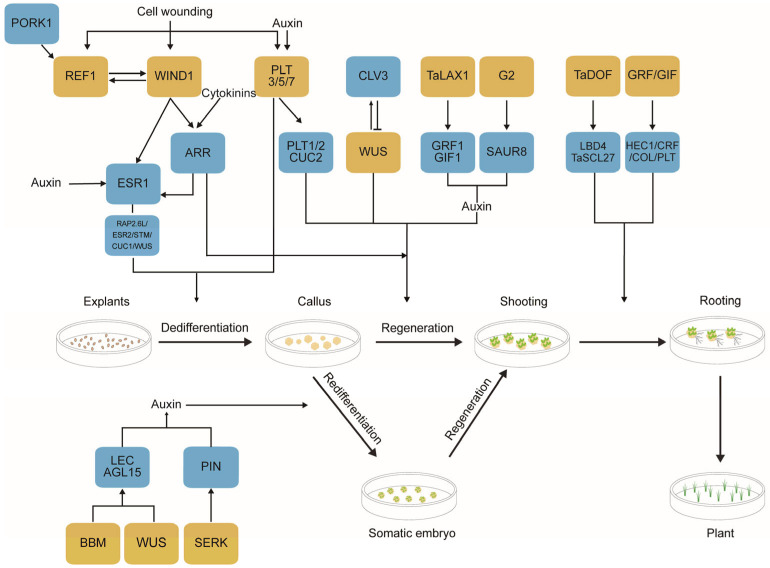
Schematic overview of DRs and their roles in enhancing plant genetic transformation. The yellow boxes highlight key developmental regulators, while the associated genes represent those specifically utilized to improve genetic transformation efficiency in this process. The blue boxes indicate downstream genes regulated by these developmental regulators. The processes depicted are exemplified by wheat (*Triticum aestivum*) transformation.

Developmental regulators (DRs) play a vital role in plant tissue culture by regulating cellular and developmental processes through transcription factors, hormones, and signaling peptides (Figure 2) [19]. Plant regeneration depends on the coordination between hormone signaling pathways and transcription factors, with DRs driving cell differentiation, proliferation, and organ formation. A deeper understanding of DRs can reveal the molecular mechanisms underlying plant regeneration and genetic transformation. This knowledge can be leveraged to optimize tissue culture protocols and overcome genotype-dependent limitations in genetic transformation, ultimately simplifying the plant GE process.

During the callus induction stage, genes such as *WIND1*, *PLT*, and *REF1* are critical. *WIND1*, an AP2/ERF transcription factor, activates downstream genes involved in cell wall remodeling and cell cycle regulation, including *ESR1*, *ESR2*, *RAP2.6L*, *CUC1*, *STM*, and *WUS*, promoting cell dedifferentiation and callus formation (Figure 2) [20]. Overexpression of *WIND1* can induce callus formation even in hormone-free mediums in crops like maize (*Zea mays*), rapeseed (*Brassica napus*), and tomato (*Solanum lycopersicum*) [21,22,23]. For example, Jiang et al. [23] discovered that *ZmWIND1* co-expression increased callus induction rates to 60.22% and 47.85% in maize inbred lines Xiang249 and Zheng58, respectively, while transformation efficiencies were 37.5% and 16.56% in the control groups. Similarly, *PLT* genes, particularly *PLT3*, *PLT5*, and *PLT7*, are essential for callus formation and bud regeneration. These genes establish cell pluripotency and regulate the pro-bud factor *CUC2* to promote bud regeneration [24]. Lian et al. [25] demonstrated that overexpression of *PLT5* enhanced genetic transformation efficiency and plant germination in *Antirrhinum majus*, tomato, rapeseed, and sweet pepper (*Capsicum annum*), with transformation efficiencies reaching 6.7–13.3%. *REF1*, a newly discovered regeneration factor, is released upon cellular damage and acts as a wound-signaling molecule. It binds to the receptor *PORK1* and activates downstream regulatory factors such as *SlWIND1*, thereby promoting callus and bud regeneration. Yang et al. [26] reported that *REF1* increased wild tomato regeneration efficiency by 5- to 19-fold and transformation efficiency by 6- to 12-fold. In wheat and maize, the application of *TaREF1* increased regeneration and transformation efficiencies in Jimai22 by 8- and 4-fold, respectively, and in maize B104 by 6- and 4-fold.

During the organ differentiation stage, auxin, cytokinin, and regulatory factors, including *WUS*, *ARR*, and *CLV,* form a complex regulatory network crucial for bud regeneration. *WUS*, a homeodomain transcription factor in the shoot apical meristem, promotes meristem formation and bud development [27]. The *CLV* gene family inhibits *WUS* to balance cell division and differentiation, while *WUS* positively regulates *CLV3* transcription, maintaining the stem cell microenvironment (Figure 2) [28]. *TaWOX5*, a *WUS* family gene in wheat, significantly improves transformation efficiency, up to 75.7–96.2% in easily transformable varieties and 17.5–82.7% in difficult-to-transform varieties. It has also been successfully applied in triticale (*Triticosecale Wittmack*), rye (*Secale cereale*), barley (*Hordeum vulgare*), and maize [29]. Additionally, *TaDOF3.4* and *TaDOF5.6* boost the expression of genes related to root and bud meristems (e.g., *TaLBD4* and *TaSCL27*), thereby enhancing wheat transformation efficiency (Figure 2). Specifically, the callus induction efficiency increased from 52% to 88% and 85%, respectively [30]. The *GRF* transcription factor family, highly conserved and plant-specific, forms a transcriptional activation complex with *GIFs* and is regulated by miR396 [31,32]. GRF and GRF-GIF fusion proteins promote cell proliferation and plant regeneration. Transgenic wheat lines containing *GRF4-GIF1* can induce green bud formation on an auxin-only medium, enabling the selection of transgenic plants without antibiotic markers [33]. Debernardi et al. [33,34] reported that *GRF4*-*GIF1* co-expression significantly enhanced wheat regeneration frequency from 2.5% to 63.0% in tetraploid wheat and from 12.7% to 61.8% in hexaploid wheat. *TaLAX1* in wheat increases regeneration efficiency by activating genes like *TaGRF4* and *TaGIF1*, thereby improving transformation and gene editing efficiency (Figure 2). Its homologs in soybean (*Glycine max*) and maize also promote cell division and regeneration [35]. *GOLDEN2* (*G2*), a GARP transcription factor regulating chloroplast development, significantly improves rice (*Oryza sativa*) transformation efficiency when a codon-optimized *rZmG2* is expressed. The average regeneration frequency of callus tissue from four rice varieties reaches 18.1–96.7% compared to control groups [36].

During the somatic embryogenesis stage, genes, including *BBM*, *WUS*, and *SERK,* play key roles (Figure 2) [37]. *BBM* activates genes related to embryo axis establishment, cotyledon formation, and embryo-specific metabolism, enhancing cell sensitivity to auxin and promoting cell division and dedifferentiation [38,39,40]. This allows somatic embryo formation on a hormone-free medium [41,42]. *WUS* and *BBM* jointly regulate the transcription of *LEC1*, *LEC2*, and *AGL15*, enhancing embryogenic ability [39]. SERK, a receptor-like protein kinase, is a key factor in somatic embryogenesis and cell signal transduction [43]. It works with PIN proteins to promote auxin synthesis and transport, increasing somatic embryo numbers [44]. Overexpression in *Arabidopsis* (*Arabidopsis thaliana*) and rice can increase somatic embryogenesis efficiency by 3–4 times and bud regeneration rates from 72% to 86%, thereby improving genetic transformation efficiency [45,46].

During the plant regeneration stage, the combined action of multiple DRs is essential for plant regeneration and genetic transformation. Simultaneous overexpression of multiple regulatory factors, such as *BBM* and *WUS*, can significantly boost transformation efficiency in difficult-to-transform species like maize, rice, and sorghum (*Sorghum bicolor*) [47,48,49]. However, ectopic expression of *BBM/WUS2* may cause developmental abnormalities and sterility in some regenerated plants [48]. Dual induction of *WIND1* and *LEC2* can enhance the formation rate of embryogenic callus in rapeseed [21].

Although the mining and application of DRs have greatly enhanced the plant transformation efficiency even without genotype limitation, it still depends on the tissue culture process, which remains challenging for the wide application of plant GE (Table 1). To overcome the transformation-based plant GE, novel strategies have been established to directly and efficiently deliver GE constructs to specific tissues, bypassing the tissue culture process, referred to as *in planta* transformation.

### 2.2. In Planta Transformation Bypassing Tissue Culture

*In planta* transformation is a genetic transformation technique that directly transforms plants in their living state rather than in vitro, eliminating the need for tissue culture processes. The floral dip method in *Arabidopsis* is a classic example [50]. Furthermore, Zhong et al. [51] introduced GUS and GE constructs by creating wounds in germinated seeds, infecting them with *Agrobacterium,* and achieving *in planta* transformation. Similarly, Wang et al. [80] developed a method for genetic transformation in *Vernicia fordii* by puncturing or infecting whole seedlings, rootless seedlings, and petioles, highlighting the importance of seedling age in determining transformation efficiency.

**Figure 3:**
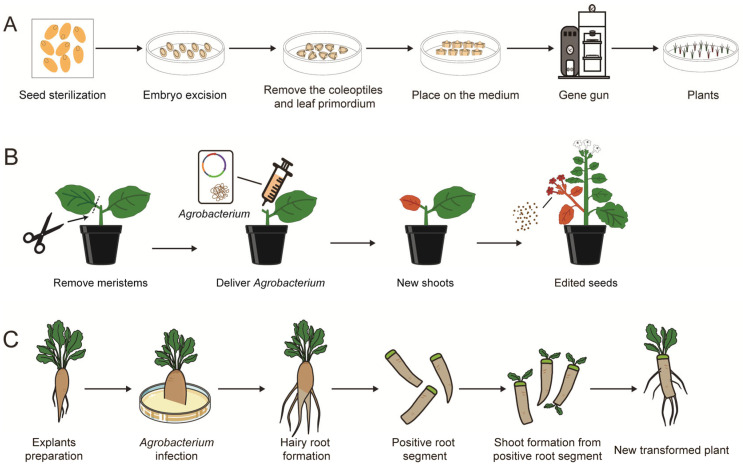
Schematic representation of *in planta* transformation bypassing tissue culture. (**A**) iPB-mediated transformation of wheat SAM via gene gun. The embryo was excised from the seed, and the SAM was exposed through dissection. After culturing the SAM in an upward orientation on a medium, it was directly bombarded using a gene gun. The SAM then underwent redifferentiation to generate new transgenic plants. (**B**) The Fast-ACC method is a rapid *Agrobacterium* co-culture technique. Apical meristems were excised from Cas9-overexpressing tobacco plants. *Agrobacterium* harboring sgRNA and DR expression vectors was introduced into the incision site. New transgenic shoots were regenerated over time, and transgenic seeds were produced on these shoots. (**C**) CDB uses *Agrobacterium* rhizogenes to induce transgenic roots from the cut sites of explants. The plant material was cut near the root and infected with *Agrobacterium* at the cut site. Subsequently, the material was directly cultured in soil. After new hairy roots emerged, the transgenic root system was excised, and shoots were regenerated from it to produce new transgenic plants.

A novel *in planta* transformation method, *in planta* particle bombardment (iPB) has been developed for wheat. This technique uses a gene gun to deliver CRISPR/Cas9 vectors directly to the shoot apical meristem (SAM) of mature seeds, enabling stable gene integration and regeneration without tissue culture (Figure 3A). It successfully created wheat *TaGASR7* knockout progenies, addressing genotype dependency issues in wheat transformation [52,53,54]. Additionally, physical means such as ultrasound can enhance the *Agrobacterium*-mediated transformation of SAM tissue to a usable level, known as SAMT, which has been applied in cotton (*Gossypium hirsutum*) [55]. However, these methods face challenges related to low editing efficiency, and further validation is required to fully assess their feasibility (Table 1).

It has been demonstrated that transient expression of DRs can induce redifferentiation in adult plants, leading to callus formation and regeneration of new plants. Maher et al. [56,57] reported a groundbreaking approach where, after removing the original meristematic tissue in tobacco (*Nicotiana benthamiana*), *Agrobacterium* was injected at the cut site to co-express the GE system and DRs. This allowed for the direct regeneration of gene-edited buds that produced flowers and seeds on the original plant, transmitting the transgenic and gene-edited traits to subsequent generations (Figure 3B). Subsequently, this method, fast-treated agrobacterium co-culture (Fast-TrACC), has been successfully applied to tomato, potato (*Solanum tuberosum*), pepper (*Capsicum chinense*), and eggplant (*Solanum melongena*), showing a certain degree of universality (Table 1) [57,58].

Building on the natural regeneration abilities of plants, Cao et al. [59] developed the cut–dip–budding (CDB) method. CDB involves cutting half of the root while retaining part of the root base tissue, infecting the cut site with *Agrobacterium*, and inducing callus regeneration and budding at that site to produce new plant materials. This approach simplifies genetic transformation steps and enables successful plant GE without tissue culture. It has been successfully validated in herbaceous plants, woody plants, and various sweet potato varieties, demonstrating its wide applicability (Figure 3C). Lu et al. [60] further tested CDB in succulent and medicinal plants, enhancing the technique by shifting the cut site from the root to the petiole and successfully inducing callus formation and *Agrobacterium*-mediated transformation at the petiole of detached leaves (Table 1). In 2024, Mei et al. [61] reported another rapid and efficient method called RAPID (Regenerative Activity-dependent *in planta* Injection Delivery), which relies on the plant’s inherent regeneration ability. This method involves injecting *Agrobacterium* at the stem node, resulting in the regeneration of transgenic adventitious roots and tuber that directly produce transgenic seedlings. RAPID has successfully been applied in sweet potatoes (*Ipomoea batatas*), potatoes and bayhops (*Ipomoea pes-caprae*) (Table 1) [61].

Another innovative approach, haploid induction editing (HI-Edit), utilizes haploid induction lines to deliver GE elements via pollen in wheat and maize. While this strategy has been demonstrated useful in *Arabidopsis*, it remains to be validated in other crops (Table 1) [62]. The grafting-mobility method involves grafting the scion of wild-type *Arabidopsis* onto transgenic *Arabidopsis* rootstocks carrying Cas9 and sgRNA. By incorporating a mobile element, the tRNA-like sequence (TLS), into the sgRNA, this technique enables heritable gene editing in the above-ground parts and seeds. Similarly, grafting the scion of wild-type *Brassica rapa* onto the rootstock of transgenic *Arabidopsis* can achieve heritable editing in *Brassica rapa*. This approach is groundbreaking for plants with limited regeneration capabilities (Table 1) [63].

The development of these *in planta* transformation methods has significantly simplified the plant GE process by overcoming the plant tissue culture process. However, their limitations in terms of efficiency, species adaptability, and scalability need to be addressed to fully realize their potential for broader application in plants (Table 1). And ongoing application and optimization of these strategies across diverse plants will undoubtedly accelerate the development of *in planta* transformation-based GE biotechnology.

### 2.3. Novel Delivery Strategy for Plant GE Constructs

**Figure 4:**
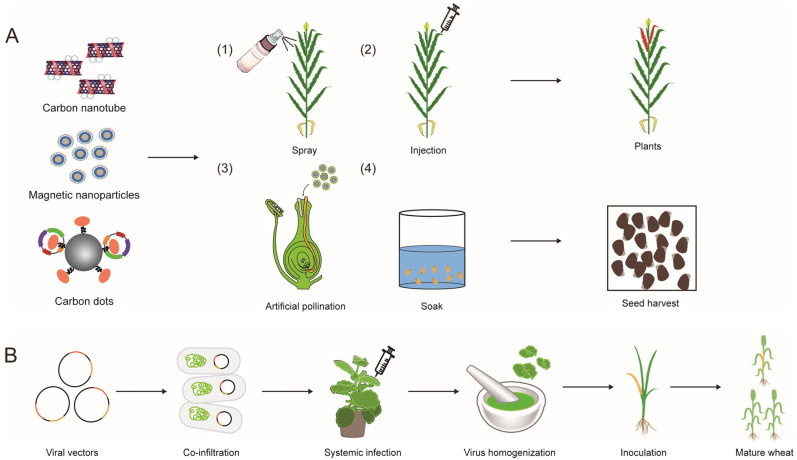
Innovative delivery strategies for plant GE constructs. (**A**) Nanomaterial-mediated delivery of GE components. Common nanomaterials used include magnetic nanoparticles, carbon nanotubes, carbon dots, and silica nanoparticles. These nanomaterials containing plasmids are delivered to various crops using different methods: (1) Carbon-based nanomaterials are uniformly mixed with a carrier to enhance the adsorption and binding of plasmids. This mixture is then sprayed onto plant leaves, allowing the nanomaterials to enter cells passively and achieve transient expression of the GE system. (2) Similar to spraying, nanomaterials can be directly injected into plant tissues to achieve GE. (3) Magnetic particles containing plasmids are mixed with pollen, and transgenic seeds can be directly obtained through the pollen tube pathway. (4) Intact rice seeds (or seeds with the shoot apical meristem exposed) can be immersed in a nanomaterial suspension, allowing nanomaterials to penetrate the seeds and produce transgenic seeds. (**B**) Virus-mediated gene editing process. *Agrobacterium* cultures harboring virus plasmids infiltrate *Nicotiana benthamiana* leaves. At 3 days post-infiltration (dpi), the infiltrated leaves are ground, and the sap is rub-inoculated onto leaves of Cas9-transgenic wheat seedlings at the two-leaf stage.

To efficiently achieve *in planta* GE across a wide range of plant species, it is essential to develop innovative delivery methods. Nanomaterials and viral delivery systems have emerged as promising tools in this regard. Nanomaterials are substances that can encapsulate molecules through non-covalent adsorption or electrostatic attraction with one or more three-dimensional dimensions reaching the nanoscale. Their application in drug delivery has been well-established in medical research. They can encapsulate proteins or nucleic acids, releasing them at specific locations to achieve transient expression. Common examples of nanomaterials include magnetic nanoparticles, carbon nanotubes (CNTs), carbon dots, and silica nanoparticles (Figure 4A) [81]. Liu et al. [64] first demonstrated that single-walled carbon nanotubes (SWCNTs) can passively pass through plant cell membranes and walls without degradation by enzymes, ushering in the era of plant nanomaterial delivery. Zhao et al. [65] successfully introduced exogenous vectors into cotton pollen using magnetic nanoparticles in the presence of a magnetic field, producing transgenic offspring that carried the exogenous genes. They also found a positive correlation between the pollen tube opening rate and the method’s efficiency [65]. Wang et al. [66] discovered that magnetic nanoparticles could efficiently deliver plasmids, with higher pollen tube opening rates observed at low temperatures (8 °C) compared to room temperature. Kwak et al. [67] utilized the pH difference between the cytoplasm (pH 5.5) and chloroplasts (pH 8.0) to release plasmids from nanomaterials in chloroplasts, using materials that only release their cargo at pH levels above 7.5. Doyle et al. [68] introduced GE constructs encapsulated by carbon dots into wheat via foliar spray and successfully achieved target gene modification, simplifying the gene editing process. Dunbar et al. [69] successfully achieved target gene modification in rice by immersing whole or partially excised seeds with exposed shoot apical meristems (SAM) in CNTs containing GE constructs.

The application of nanomaterials in plant GE offers several significant advantages, including independence from plant genotypes and the lack of genome integration. This holds particular promise for crops like cassava (*Manihot esculenta Crantz*), cacao (*Theobroma cacao*), and sugarcane (*Saccharum officinarum*), which are unable to achieve non-GMO gene editing through traditional hybridization methods. However, there are challenges associated with the use of nanomaterials [81]. High costs, low transformation efficiency, and concerns about the potential non-degradability of delivery materials currently limit their widespread practical application (Table 1). These issues necessitate further research and development to enhance the feasibility and effectiveness of nanomaterial-based GE in plants.

Viral delivery systems utilize the inherent capability of viruses to transport nucleic acid. Engineered viral vectors, capable of replication, self-assembly, and movement between plant cells, are designed to deliver exogenous DNA, such as RNAi and GE reagents, to plant cells. Plant viruses used mainly include positive-strand RNA viruses (PSVs), negative-strand RNA viruses (NSVs), single-stranded DNA viruses, and double-stranded DNA viruses. RNA viruses, being the majority of plant viral pathogens, account for 90% of crop losses elicited by virus diseases [82]. Thus, several RNA viruses have been engineered for GE in both monocot and dicot plants (Figure 4B) [83,84,85,86,87,88,89]. However, the capacity of viruses is limited, necessitating the use of Cas-overexpressing transgenic plants for virus-mediated gene editing. Additionally, GE events in plants are often not inheritable (Table 1).

Ellison et al. [70] first reported that the *Tobacco Rattle Virus* (TRV) vector delivering fused sgRNA with flowering locus T (FT) RNA or tRNA could generate the edited progeny in Cas9-overexpressing tobacco. The mobile RNA elements promote the cell-to-cell mobility of sgRNA, which finally moves into meristematic tissues, directly inducing inheritable plant GE and bypassing the tissue culture process. This method was later validated in *Arabidopsis* and tomato [71,72]. Two other research studies showed its application in wheat using *Barley Stripe Mosaic Virus* (BSMV) to generate GE progeny in Cas9-overexpressing lines [73,74]. Although this strategy has been demonstrated useful in other viruses, it remains limited depending on the Cas9-overexpressing plants. Another heritable method involves leaf passage after virus inoculation. For example, *Tomato Spotted Wilt Virus* (TSWV), a negative-sense RNA virus, can infect the entire plant from inoculated leaves. By cultivating leaves showing viral infection symptoms on a non-selective medium, efficient and short-term heritable gene editing can be achieved. This method has been successfully applied to crops like tobacco, tomato, and pepper [75,76].

Geminiviruses, single-stranded DNA (ssDNA) viruses, offer superior cargo capacity and high recombinant gene expression, making geminiviral replicons a suitable vector for delivering CRISPR components along with donor DNA for precise genome editing through homology-directed repair (HDR) [77,78]. Although Geminiviruses-mediated GE has enabled highly efficient and precise gene modification, it still depends on the traditional *Agrobacterium*-based transformation to generate GE plants. Recently, Weiss et al. [79] utilized a TRV vector to deliver the compact nuclease TnpB alongside ωRNA and successfully achieved heritable editing in *Arabidopsis*. Additionally, other compact nucleases, such as Cas12f and CasΦ, have been identified and validated for their functionality in major crops like rice and wheat [90,91]. These nucleases hold significant promise for delivery into plants via viral vectors. With continuous discovery and engineering of novel virus vectors, it is expected to expand to more crops, overcoming the transformation limitations and genotype constraints (Table 1).

## 3. Concluding Remarks and Future Perspectives

The rapid advancement of GE technologies has significantly transformed plant research and breeding. However, traditional genetic transformation methods are often hindered by genotype dependency and the labor-intensive tissue culture process. This review has highlighted several innovative strategies to simplify plant GE by enhancing tissue culture efficiency, bypassing tissue culture through *in planta* transformation, and employing novel delivery methods such as nanomaterials and viral vectors. The use of DRs has shown considerable promise in enhancing tissue culture efficiency by optimizing the regeneration process and overcoming genotype limitations. *In planta* transformation methods, such as iPB, Fast-TrACC, and CDB, have demonstrated the potential to completely bypass tissue culture altogether, significantly simplifying the GE process. Additionally, the application of nanomaterials and viral vectors offers genotype-independent and tissue culture-free alternatives for delivering GE constructs, although challenges such as high costs and low efficiency still persist (Table 1).

Looking to the future, several key areas warrant further exploration and development. First, a deeper understanding of the molecular mechanisms underlying plant regeneration and the role of developmental regulators will provide valuable insights for further improving tissue culture efficiency and overcoming genotype barriers. Second, the optimization and application of *in planta* transformation methods across a broader range of plant species will be crucial for widespread adoption. This includes addressing the current limitations of these methods, such as low editing efficiency and potential developmental abnormalities in regenerated plants. Third, the development of more efficient and cost-effective nanomaterials and viral vectors will be essential for practical application in diverse crops, especially those with limited regeneration capabilities or that are difficult to transform using traditional methods. Moreover, the integration of these innovative strategies with existing GE technologies, such as base editors and prime editors, will enhance the precision and versatility of plant genome editing. Finally, the regulatory landscape for the commercialization of GE crops varies greatly across countries, largely due to the ongoing debate surrounding genetically modified organisms (GMOs). In regions including the European Union, while research on GE crops is allowed, their commercialization is subject to strict restrictions [92,93]. Therefore, the convergence of novel delivery materials and plant regeneration methods heralds a new era of one-step GE without DNA integration, effectively addressing the regulatory concerns associated with GE crops in agriculture.

In summary, the ongoing development and optimization of simplified plant GE strategies hold great potential for accelerating plant research and breeding, ultimately contributing to global food security and sustainable agriculture. Future work should focus on overcoming the remaining challenges and leveraging the strengths of these innovative approaches to unlock the full potential of plant genetic engineering.

## Figures and Tables

**Table 1:** A comprehensive summary of simplified plant GE strategies.

	Name of Strategy	Advantages	Disadvantages	Examples for Application	References
Optimization of tissue culture process through developmental regulators	--	High efficiency; no genotype limitation	Reliance on tissue culture; time-consuming; labor-intensive	*Oryza sativa*/ *Zea mays*/ *Triticum aestivum*/ *Solanum lycopersicum*/*Sorghum bicolor*/ *Arabidopsis thaliana*/ *Glycine max*/ *Nicotiana benthamiana*/*Brassica napus*/ *Coffea canephora*/ *Cannabis sativa*	[20,21,22,23,24,25,26,27,28,29,30,31,32,33,34,35,36,37,38,39,40,41,42,43,44,45,46,47,48,49]
*In planta* transformation bypassing tissue culture	Floral dip	Easy operation; high efficiency; no genotype limitation; independence of tissue culture	Limitation to different plant species	*Arabidopsis thaliana*	[50,51]
	iPB, SAMT	Independence of tissue culture	Relatively low efficiency; genotype limitation; requirement of further validation of more plant species	*Triticum aestivum*/*Gossypium hirsutum*	[52,53,54,55]
	Fast-TrACC	Easy operation; high efficiency; independence of tissue culture	Genotype limitation; requirement of further validation of more plant species	*Nicotiana benthamiana*/*Solanum lycopersicum*/*Solanum tuberosum*/ *Capsicum annuum*/ *Solanum melongena*	[56,57,58]
	CDB	Relatively high efficiency; independence of tissue culture; no genotype limitation	Limited regeneration capacity; requirement of further validation of more plant species	*Taraxacum kok-saghyz*/ *Coronilla varia*/ *Ailanthus altissima*/ *Aralia elata*/ *Clerodendrum chinense*/ *Ipomoea batatas*/ *Kalanchoe blossfeldiana*/ *Crassula arborescens*/ *Sansevieria trifasciata*	[59,60]
	RAPID	Relatively high efficiency; independence of tissue culture	Genotype dependence; high technical requirements; requirement of further validation of more plant species	*Ipomoea batatas*/ *Solanum tuberosum*/ *Ipomoea pes-caprae*	[61]
	HI-Edit	Independence of tissue culture	Relatively low efficiency; high technical requirements; requirement of further validation of more plant species	*Zea mays*/ *Triticum aestivum*/ *Arabidopsis thaliana*	[62]
	Grafting-mobility	Relatively high efficiency; independence of tissue culture	Genotype dependence; requirement of further validation of more plant species	*Arabidopsis thaliana*/ *Brassica napus*	[63]
Novel delivery platforms	Nanoparticle	Easy operation; no genotype limitation; independence of tissue culture	Low efficiency; high cost; requirement of further validation of more plant species	*Triticum aestivum*/ *Gossypium hirsutum*/ *Zea mays*/*Oryza sativa*/ *Nicotiana benthamiana*/	[64,65,66,67,68,69]
	Viral vector	High efficiency; easy operation; no genotype limitation; independence of tissue culture	Limited viral vector capacity; potential biosafety issues; requirement of further validation of more plant species	*Triticum aestivum*/ *Nicotiana benthamiana*/*Solanum tuberosum*/ *Capsicum*/ *Arabidopsis thaliana*/ *Gossypium hirsutum*	[70,71,72,73,74,75,76,77,78,79]
